# An Automated Machine-Learning Approach for Road Pothole Detection Using Smartphone Sensor Data

**DOI:** 10.3390/s20195564

**Published:** 2020-09-28

**Authors:** Chao Wu, Zhen Wang, Simon Hu, Julien Lepine, Xiaoxiang Na, Daniel Ainalis, Marc Stettler

**Affiliations:** 1 School of Public Affairs, Zhejiang University, Hangzhou 310058, China; chao.wu@zju.edu.cn; 2 College of Software Engineering, Zhejiang University, Hangzhou 310058, China; harvien9@gmail.com; 3 ZJU-UIUC Institute, School of Civil Engineering, Zhejiang University, Haining 314400, China; 4 Department of Operations and Decision Systems, Université Laval, Quebec City, QC G1V 0A6, Canada; julien.lepine@fsa.ulaval.ca; 5 Department of Engineering, University of Cambridge, Trumpington Street, Cambridge CB2 1PZ, UK; xnhn2@cam.ac.uk (X.N.); dta32@eng.cam.ac.uk (D.A.); 6 Department of Civil and Environmental Engineering, Imperial College London, London SW7 2AZ, UK; m.stettler@imperial.ac.uk

**Keywords:** road quality monitoring, shock detection, pothole detection, crowdsourced data, support vector machine, random forest

## Abstract

Road surface monitoring and maintenance are essential for driving comfort, transport safety and preserving infrastructure integrity. Traditional road condition monitoring is regularly conducted by specially designed instrumented vehicles, which requires time and money and is only able to cover a limited proportion of the road network. In light of the ubiquitous use of smartphones, this paper proposes an automatic pothole detection system utilizing the built-in vibration sensors and global positioning system receivers in smartphones. We collected road condition data in a city using dedicated vehicles and smartphones with a purpose-built mobile application designed for this study. A series of processing methods were applied to the collected data, and features from different frequency domains were extracted, along with various machine-learning classifiers. The results indicated that features from the time and frequency domains outperformed other features for identifying potholes. Among the classifiers tested, the Random Forest method exhibited the best classification performance for potholes, with a precision of 88.5% and recall of 75%. Finally, we validated the proposed method using datasets generated from different road types and examined its universality and robustness.

## 1. Introduction

Road defects, such as potholes and cracks, are becoming an increasingly significant problem for roads around the world. They present a hazard for all road users, causing considerable vehicle damage. In the US alone, one in three drivers experiences pothole-induced damage to their vehicle, spending >3 billion US dollars annually for repairs [1]. Consequently, the damage induced by potholes has resulted in expensive lawsuits and damage claims [2]. Despite the large government investment made in maintaining and repairing road infrastructure, few people are satisfied with the quality of roads where they live or work [3].

Maintaining high-quality road infrastructure is challenging for numerous reasons, including harsh weather, unexpected road loads, and inconsistent wear and tear. As road damage and normal wear are highly unpredictable, an infrastructure maintenance program is only as effective as its associated monitoring program. For instance, traditional pothole detection methods utilize dedicated inspection vehicles to conduct routine checks [4,5,6]. This is expensive, and many local authorities presently face significant budgetary constraints, resulting in less frequent inspections that are only able to cover limited portions of road networks. Meanwhile, the popularity and ubiquity of smartphones provide an opportunity to collect data from crowds. The built-in accelerometer and global positioning system (GPS) can measure the road surface quality and detect potholes [7,8].

**Figure 1:**
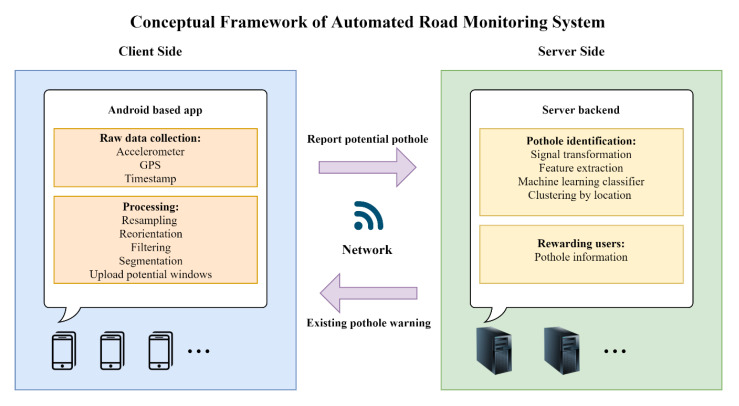
Proposed system architecture.

We propose a conceptual framework for an automated pothole detection system using vibration data acquired from smartphones, as shown in Figure 1. A mobile phone application needs to be installed on the user’s mobile phone to collect the vibration signal and location information. The app also implements some necessary processing (e.g., resampling, reorientation, filtering, etc.) on the collected data, and then divides the continuous signal into segments with a sliding window. Meanwhile, potential pothole-related segments are identified using simple thresholds and reported to the server together with their GPS location through the mobile communication network. The server back-end extracts features from the uploaded data after signal transformation, and identifies real potholes by using pretrained machine-learning classifiers. Potholes identified from multiple vehicles’ data are clustered to determine the final pothole using the clustering method. The final potholes are saved to a custom database that can be used by a road maintenance department. It is possible to use this app to provide information on approaching potholes to remind drivers to slow down and be careful, as a reward for using the app. In this conceptual framework, we prove the feasibility of the system through an offline simulation, include all steps of data processing and pothole identification (except for clustering by location).

The following section provides a literature review of state-of-the-art road condition monitoring approaches using mobile devices, the identified gap in the literature, and our proposed solution. Section 3 introduces the detailed pipeline of our proposed method. We analyze and discuss the experimental results in different circumstances in Section 4, and Section 5 summarizes the work presented in this paper and discusses future research directions.

## 2. Literature Review

Existing methods for the monitoring of road conditions using mobile devices are mainly based on camera observations and vibration detection. Vision-based methods rely on mobile devices mounted on a driving vehicle to capture pictures of the road surface, and automatically analyze the road information contained in the pictures through image analysis algorithms. For example, the Video-based Pavement Distress Screening (VPADS) [9] system detects road distress by applying an automatic data processing workflow to video data. Large image datasets of road surfaces were built by Maeda et al. [10] and Ochoa-Ruiz et al. [11] for the effective detection of road damage using deep learning algorithms. In vibration-based methods, accelerometers and gyroscopes are the most commonly used sensors, as they are sensitive to shocks induced by road anomalies (e.g., potholes and bumps), along with the GPS, which is used to record location. In this paper, vibration-based methods are the main focus of our research. Many methods have been used, which can be divided into the following three categories: (1) threshold-based methods, (2) dynamic time warping (DTW), and (3) machine learning together with feature engineering [12]. The use of clustering results from various users has become an effective postprocessing approach that significantly improves the successful detection rate and reduces the false positive rate [12].

Threshold-based approaches identify anomalies when the amplitude or other properties of the signal (e.g., root mean square (RMS) and crest factor) exceed a specified value. Nericell [13] proposed a system to find potholes and bumps utilizing two detectors depending on the speed of the vehicle. Virtual reorientation is implemented in advance to make the disoriented accelerometer have almost the same orientation as the vehicle coordinate system for monitoring the road conditions. At high speeds, a spike (or change point detection) along $a_{z}$ above the specified threshold is classified as a suspected anomaly, while the detector searches for a sustained dip in $a_{z}$ that is produced when the tires enter the pothole at low speed. Over several observations, the detector maintained a low false positive rate (5–10%), but a high false negative rate (20–30%). Mednis et al. [14] suggested that all three-axis acceleration data were close to 0 g when the vehicle was in temporary freefall while entering or exiting the potholes. Therefore, they proposed a G-ZERO algorithm for detecting potholes and compared it with three other threshold-based methods: Z-THRESH, Z-DIFF, and STDEW(Z). The threshold and sliding window size were changed for the tuning of each algorithm for pothole detection, and the best true positive rate achieved was 90%. A system named TERM [15] is a road monitoring system using dynamic thresholds and crowdsourcing methods. In this algorithm, a potential pothole is detected when the acceleration values of the *X*- (lateral) and *Z*-axes (vertical) exceed the specified threshold, and a speed bump is detected when the acceleration values of the *Y*- (longitudinal) and *Z*-axes exceed the specified threshold. When the same location is identified as a potential anomaly (pothole or speed breaker) by more than five data samples, it is identified as an actual anomaly and marked on the map. Although the detection algorithms are simple, they achieve good results, i.e., the successful detection of 90% of speed breakers and 85% of potholes, primarily due to the use of crowdsourcing data. Despite the positive results, the false positive rate of the system was not disclosed.

DTW is a pattern-matching algorithm for measuring the similarity between two temporal sequences, which may vary in time and space [12]. Singh et al. [16] utilized accelerometer sensor data to detect road anomalies and distinguish specific types of anomalies using the DTW technique. Reference templates of potholes and bumps were manually derived from accelerometer data and stored in a template database. Detection was performed by calculating the similarity value between the input data and the reference templates. The detection rate of the system was 88.7% and 88.9% for potholes and bumps, respectively.

Machine-learning techniques have been widely adopted in road inspection. Pothole Patrol [17] collected data using mobile sensors (accelerometer, GPS) installed on vehicles that travelled thousands of kilometers around the city of Boston, MA, USA. In their pothole-detection algorithm, a range of filters (speed, high-pass, *z*-peak, *xz*-ratio, speed vs. *z*-ratio) were applied to dismiss one or more nonpothole event types. The thresholds of each filter are used as the tuning parameters to optimize the system’s detection accuracy, which is consistent with the basic idea of machine-learning. By clustering the results according to location, the system achieved a final pothole-detection accuracy of 92.4% for labelled data. In contrast to this Pothole Patrol ($p^{2}$) system, in most machine-learning methods for road inspection, the following processing steps are generally adopted. First, features of different domains are extracted from the preprocessed data via various approaches. Then, classification is applied to these features to identify road defects, and different types of anomalies are distinguished [18]. Perttunen et al. [19] extracted features from the time and frequency domains using the fast Fourier transform (FFT), and the feature selection algorithm was subsequently utilized to select the optimal feature sets. Because features are affected by speed, Perttunen et al. eliminated the linear dependency on speed by fitting a line to each feature of the data. Seraj et al. [20] not only investigated the time and frequency domains, but also took features from wavelet transformation into account by employing the stationary wavelet transform, which is a type of nondecimated discrete Wavelet transform (DWT). Both Perttunen et al. [19] and Seraj et al. [20] used a support vector machine (SVM) as the classification model. Silva et al. [21], Varona et al. [22], Lepine et al. [23], and Basavaraju et al. [24] analyzed and evaluated different classifiers. Borrowing ideas from the field of computer vision, Varona et al. augmented datasets by stretching and shrinking the sequence of variation signals. Basavaraju et al. examined the differences between using features from all axes and features from the *Y*-axis only to investigate the possibility of using single-axis data. Li et al. [25] were the first to apply the continuous Wavelet transform to feature extraction in pothole detection; they proposed a novel solution for estimating the pothole size.

Compared to threshold-based methods and the DWT, machine-learning is a more advanced and comprehensive method that is able to extract more useful information from the data. Although machine-learning techniques are mature and yield good results, the threshold is still the basis of this approach; i.e., the machine-learning is simply used to transfer the process of finding suitable thresholds from humans to machines. However, in previous studies, researchers often overlooked the fact that most of the original data are induced by flat road sections, which can be removed by simple thresholds. In most studies, machine-learning classifiers were directly implemented without the application of any prediscarding filters. This not only increased the power and data consumption—because the client uploaded redundant data—but also increased the burden on the server for machine-learning classification. Another limitation identified is that the road type was not considered during pothole detection. A model trained on a dataset with a certain road type (such as a highway) may not be suitable for another road type, because the occurrence frequencies, shapes, and sizes of potholes differ.

The contribution of this paper are as follows:

- A pothole-detection approach using smartphones is proposed and verified via experiments, including a series of data-processing methods. A feasible algorithm is developed for calculating the yaw angle of reorientation, and thresholds are used to select potential pothole windows, which are not mentioned in most related works.
- The performance for different feature domains is analyzed and compared with regard to the time required and the pothole-detection accuracy. The strengths of various classifiers are examined, and applicable scenarios for the classifiers are suggested.
- The performance of the proposed method for datasets generated from different types of roads is assessed to evaluate its universality and robustness. The pothole-detection capability of our method is better than that of most previously reported methods.

## 3. Methodology

**Figure 2:**
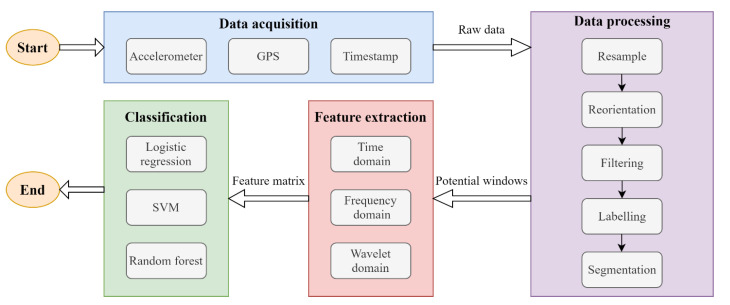
Workflow of the proposed methodology.

The objective of this study is to develop a method for detecting potholes using vibration sensors embedded in smartphones. We propose a useful crowdsourced automated road-monitoring system, as shown in Figure 1. The general workflow consists of four stages: (1) data acquisition, (2) data processing, (3) feature extraction, and (4) classification (as shown in Figure 2). First, data acquisition is performed to collect vibration information for the running vehicle. Second, a series of data-processing methods are used to process the raw data collected in the first stage, and sliding windows together with simple threshold methods are utilized to identify potential potholes. Third, features of different domains are extracted from potential pothole windows after a series of signal transformations. Finally, supervised machine-learning classifiers are applied for training to distinguish road defects. Except for the data acquisition, all processing was conducted offline using a laptop.

### 3.1. Data Acquisition

**Figure 3:**
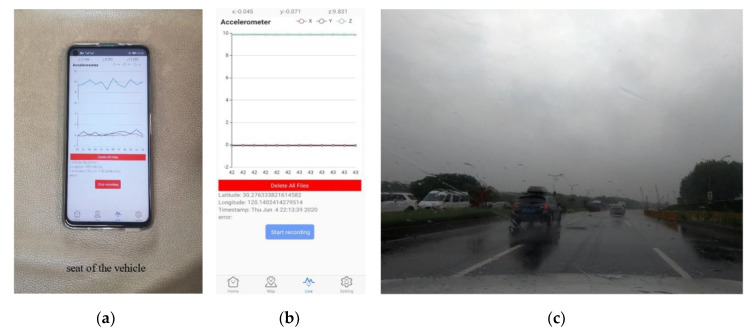
(**a**) Placement of the phone, (**b**) screenshot of the data-collection application and (**c**) scene of our experimental site recorded by the video camera.

Nericell [13] demonstrated that the built-in accelerometer of smartphones could capture the shock induced by road anomalies. Using the embedded GPS chip, this shock can be tagged with location information, allowing road anomalies to be recorded on a digital map. In the present study, a mobile network-connected smartphone with a triaxial accelerometer and a GPS chip was employed. A customized Android application (as shown in Figure 3b) was developed to collect data from these sensors. The accelerometer had a sampling rate of 50 Hz, whereas the GPS sampling rate was only 1 Hz, owing to the device limitations. For convenience, the continuous sequence signals were uploaded to a real-time database provided by Firebase [26] and then downloaded for analysis and processing offline.

**Figure 4:**
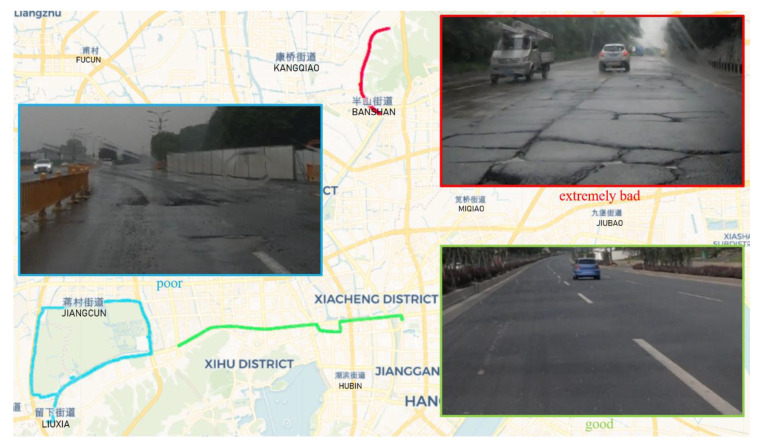
Experimental driving trajectory and corresponding road conditions. The red line above shows one of multiple trajectories driving on a good road section.

We used a five-seater Shanghai-Volkswagen Lavida Sedan (Volkswagen, Beijing, China) as the experimental vehicle and a video camera was attached to the windshield to record the road conditions (as shown in Figure 3c). The smartphone was placed on the back seat without fixing (as shown in Figure 3a), since the back seat is close to the center of the car, in which the vibration signal is more representative. The data-collection experiment was conducted on various roads in Hangzhou City (China), as shown in Table 1, during a relatively low traffic period, with a constant driving speed of about 30 ± 5 km/h. Based on the observational data collected during this study, we divided the road quality into three categories: ‘good’, ‘poor’ and ‘extremely bad’ (as shown in Figure 4). It should be noted that these categories are compared relatively rather than absolutely. For example, the quality of urban roads was poor; there were defects on the roads because a subway was under construction nearby. The quality of suburban roads was extremely bad, with many potholes, owing to a lack of maintenance; furthermore, large trucks used the roads throughout the year. We also performed experiments on elevated roads and national roads of good quality, but during the subsequent data processing, we found that these road sections had almost no potholes; thus, they were excluded from the analysis.

### 3.2. Data Processing

Data processing is a prerequisite for obtaining useful features for analysis. In this research, the data processing was divided into five steps (as shown in Figure 2): resampling, reorientation, filtering, labelling, and segmentation.

#### 3.2.1. Resampling

**Figure 5:**
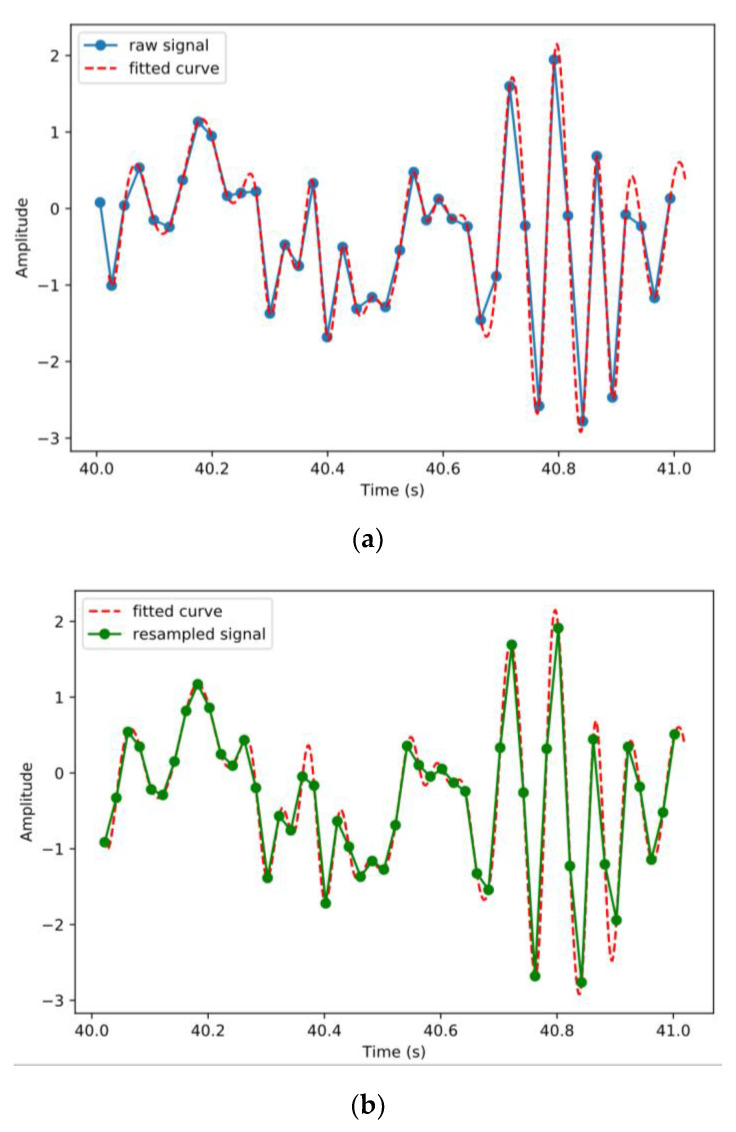
Illustration of the resampling process showing a sample of (**a**) the raw acceleration signal and its fitted curve; (**b**) the fitted curve and the resampled signal.

During the experiments, the smartphone was found to be unable to uniformly sample the accelerometer data at the designated frequency. The sampling rate set for the accelerometer in the app was 50 Hz, but analysis revealed that the accelerometer only captured approximately 43 to 47 samples per second. The variable sampling rate made the time interval between samples inconsistent, presenting a problem: the Fourier transform could not be performed on the signal sequence. Therefore, we resampled the signal at 50 Hz by interpolating the data uniformly. The Scipy [27] library of Python provides a useful interpolation function. Specifically, scipy.interpolate.splrep() fits a B-spline one-dimensional curve for the given discrete points, as shown in Figure 5a, and then scipy.interpolate.splev() samples uniform points from the fitted curve, as shown in Figure 5b. The resampling frequency was set as 50 Hz because the formerly designed sampling rate was 50 Hz.

#### 3.2.2. Reorientation

**Figure 6:**
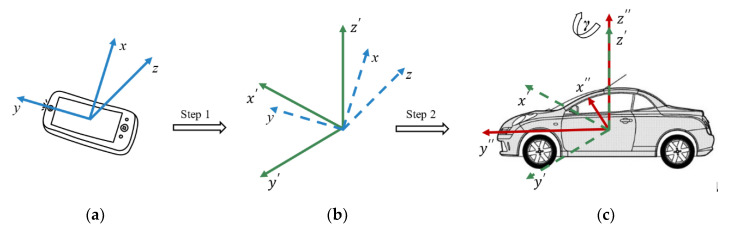
(**a**) Cartesian coordinate axes of the smartphone accelerometer; (**b**) alignment of the *z*-axis of the data with that of the vehicle; (**c**) alignment of the *X*- and *Y*-axes of the data with those of the vehicle.

The built-in triaxial accelerometer sensor detects linear acceleration on three axes by measuring inertial forces, which can be represented as $a_{x}$, $a_{y}$, and $a_{z}$. Its default coordinate system relative to the smartphone frame is shown in Figure 6a. In general, the smartphone is not ideally placed to coincide with the reference coordinate system of the vehicle, as shown in Figure 6c. Therefore, for the rationality and compactness of the research, reorientation was performed to transfer acceleration data from the smartphone coordinate system to the vehicle coordinate system. Euler angles facilitated this transformation [28]. The Euler angles included the roll angle *α*, pitch angle β, and yaw angle γ. By sequentially rotating *α*, β, and γ around the *X*-, *Y*-, and *Z*-axes, respectively, the data value in one coordinate system was transferred to another, in accordance with Equation (1). Because the calculation of γ depends on detecting the acceleration or deceleration events of the running vehicle, which requires that the *z*-axis of the acceleration data coincide with that of the vehicle, the reorientation was performed in two steps, as shown in Figure 6. In the first step, the *z*-axis of the data was aligned with that of the vehicle, and in the second step, the *X*- and *Y*-axes were aligned with those of the vehicle [29].

(1) $$\left[\begin{array}{c} a_{x}^{\prime \prime }\\ a_{y}^{\prime \prime }\\ a_{z}^{\prime \prime } \end{array}\right]=R_{z}\left(\gamma \right)R_{y}\left(\beta \right)R_{x}\left(\alpha \right)\left[\begin{array}{c} a_{x}\\ a_{y}\\ a_{z} \end{array}\right]=\left[\begin{array}{ccc} cos\gamma & -sin\gamma & 0\\ sin\gamma & cos\gamma & 0\\ 0 & 0 & 1 \end{array}\right]\;\left[\begin{array}{ccc} cos\beta & 0 & sin\beta \\ 0 & 1 & 0\\ -sin\beta & 0 & cos\beta \end{array}\right]\;\left[\begin{array}{ccc} 1 & 0 & 0\\ 0 & cos\alpha & -sin\alpha \\ 0 & sin\alpha & cos\alpha \end{array}\right]\left[\begin{array}{c} a_{x}\\ a_{y}\\ a_{z} \end{array}\right]$$

According to Newton’s law, when a vehicle is stationary or moving in a straight line at a constant speed on a level surface, the measured acceleration expressed in the vehicle coordinate system is given by Equation (2).

(2) $$a_{x}^{\prime \prime }=0\;;\;a_{y}^{\prime \prime }=0\;;\;a_{z}^{\prime \prime }=1g$$

Two of three Euler angles can be calculated from this condition using Equations (3) and (4) [7]. The roll angle *α* is in the range [-π; π], and the pitch angle *β* is in the range [-π/2; π/2] [30].

(3) $$\alpha =\;tan^{-1}\left(\frac{a_{y}}{a_{z}}\right)$$

(4) $$\beta =\;tan^{-1}\left(\frac{-a_{x}}{\sqrt{a_{y}^{2}+a_{z}^{2}}}\right)$$

After α and β are calculated, the first-step reoriented accelerations along three axes can be estimated using Equation (5).

(5) $$\left[\begin{array}{c} a_{x}^{\prime }\\ a_{y}^{\prime }\\ a_{z}^{\prime } \end{array}\right]=\;R_{y}\left(\beta \right)R_{x}\left(\alpha \right)\left[\begin{array}{c} a_{x}\\ a_{y}\\ a_{z} \end{array}\right]=\;\left[\begin{array}{ccc} cos\beta & 0 & sin\beta \\ 0 & 1 & 0\\ -sin\beta & 0 & cos\beta \end{array}\right]\;\left[\begin{array}{ccc} 1 & 0 & 0\\ 0 & cos\alpha & -sin\alpha \\ 0 & sin\alpha & cos\alpha \end{array}\right]\left[\begin{array}{c} a_{x}\\ a_{y}\\ a_{z} \end{array}\right]$$

With the *z*-axis of the 3D acceleration data aligned with the vehicle, the yaw angle γ can be estimated from the dynamic condition. When the vehicle is braking or accelerating rapidly in a straight line, it experiences considerable acceleration in the driving direction, with no acceleration in the other horizontal directions. Therefore, we utilize such a deceleration or acceleration event and take the direction of the component of the first-step reoriented acceleration data on the horizontal plane as the driving direction of the vehicle, to establish the relationship between the acceleration data and the driving direction of the vehicle. Starting from this condition, the yaw angle γ can be calculated using Equation (6). It is in the range [−π; π].

(6) $$\gamma =\;tan^{-1}\left(\frac{a_{x}^{\prime }}{a_{y}^{\prime }}\right)$$

After γ is calculated, the second-step reoriented accelerations along the three axes can be estimated using Equation (7).

(7) $$\left[\begin{array}{c} a_{x}^{\prime }\\ a_{y}^{\prime }\\ a_{z}^{\prime } \end{array}\right]=R_{z}\left(\gamma \right)\left[\begin{array}{c} a_{x}^{\prime }\\ a_{y}^{\prime }\\ a_{z}^{\prime } \end{array}\right]=\left[\begin{array}{ccc} cos\gamma & -sin\gamma & 0\\ sin\gamma & cos\gamma & 0\\ 0 & 0 & 1 \end{array}\right]\;\left[\begin{array}{c} a_{x}^{\prime }\\ a_{y}^{\prime }\\ a_{z}^{\prime } \end{array}\right]$$

**Figure 7:**
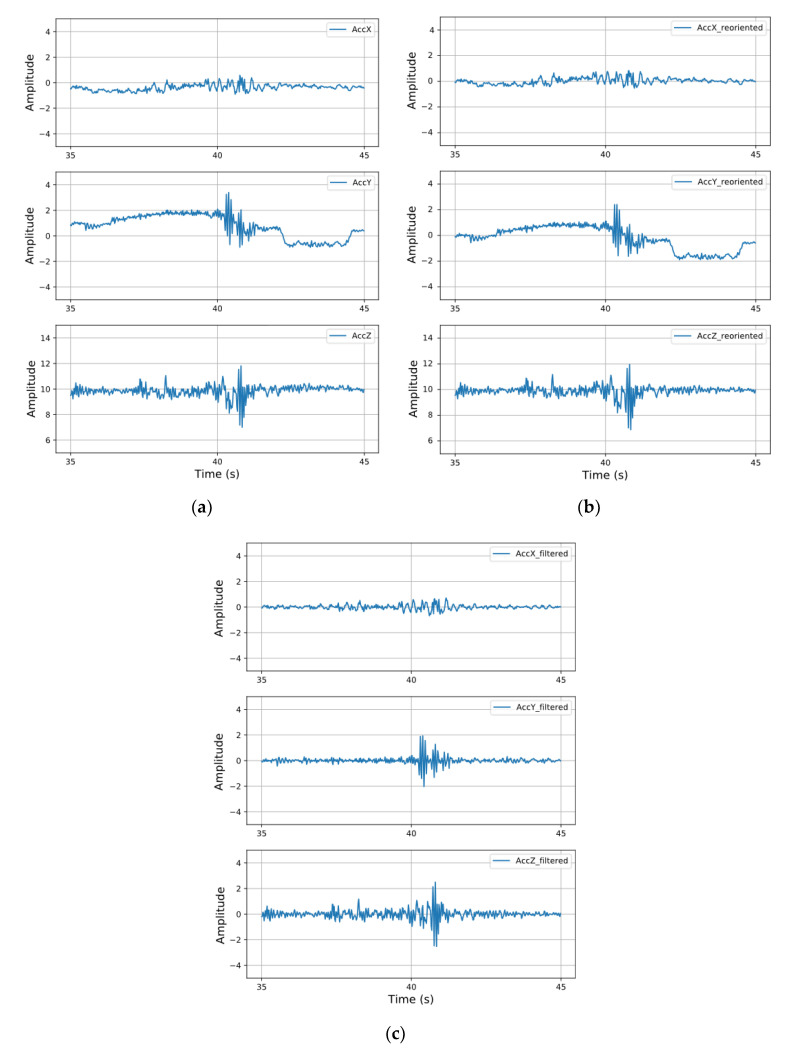
(**a**) Acceleration signals along the three axes before reorientation; (**b**) acceleration signals after reorientation; (**c**) acceleration signals after reorientation and filtering.

The proposed algorithms for the two steps of the reorientation are presented in the Appendix A as Algorithms A1 and Algorithm A2. After all the reorientation steps, we calculate the average acceleration values of all the samples and find that the acceleration of the *z*-axis is close to 1 g, while those of the other two axes are close to 0. This indicates that the *z*-axis of the reoriented acceleration data is perpendicular to the ground, which validates Algorithm A1. We also validate Algorithm A2 by randomly selecting consecutive samples that have evident negative *Y*-axis values along with zero *X*-axis values. In the corresponding period, we observed that the vehicle in the video was in a process of deceleration, proving the correctness of Algorithm A2. Figure 7a,b show plots of the acceleration data along the three axes before and after the reorientation, respectively. Particularly, all subsequent data processing steps are based on reoriented data.

#### 3.2.3. Filtering

To exclude driving conditions that are unrelated to the road quality, such as the vehicle acceleration, deceleration, and turning, the reoriented signal along three axes must be filtered. In this study, an 11th-order Butterworth high-pass filter (as recommended in Basavaraju [24]) with a cut-off frequency of 2 Hz is used, which removes the low-frequency component caused by the acceleration and deceleration of the running vehicle while retaining the high-frequency component of the signal caused by road surface anomalies. Plots of the acceleration data along the three axes before and after the filtering are presented in Figure 7b,c, respectively. As indicated by the *Y*-axis data, the filter eliminated the component caused by the speed change of the vehicle, and simultaneously, the gravity component was removed by observing the changes in the *Z*-axis data.

#### 3.2.4. Labelling

**Figure 8:**
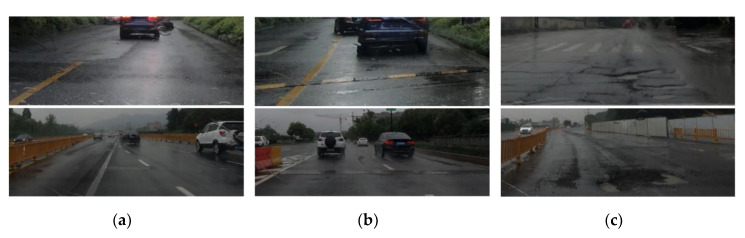
Three categories of road surface: (**a**) normal: smooth road, flat manholes, slight cracks, holes, patches and a series of tiny defects, (**b**) transverse: long transverse cracks, speed bumps, road joints, etc. and (**c**) pothole: deep-sunk manholes, severe cracks, holes and thick-protruding patches, etc.

Labelling was required for research purposes, i.e., so that a supervised learning model could be trained and the performance of the algorithm could be evaluated. In this study, the filtered data are labelled manually by using the recorded video footage as the ground truth. We find that most transversal anomalies such as speed bumps, road joints, etc. do not need to be repaired. Therefore, in this study, transverse and pothole are separated, resulting in the following three categories, as shown in Figure 8.

**Figure 9:**
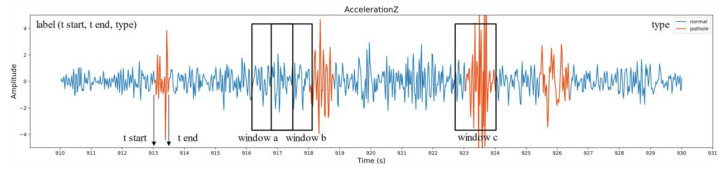
Labelled acceleration sequence. Four pothole events are presented, along with three different types of windows.

The start time, end time, and types of road anomalies (transverse or pothole) are the information to tag, resulting in a tuple (start time, end time, type) for every anomaly. The signal peak and road conditions in the video together serve as the basis for labelling anomalies to ensure that small anomalies and anomalies that the wheel does not pass are not mislabeled. The labelled acceleration sequence is shown in Figure 9, and the pothole events are highlighted in orange. The manual labels are tagged to windows in the next procedure, which is called segmentation.

#### 3.2.5. Segmentation

For the choice of sliding window, we want to select a window covering approximately 10 m traveling distance. Assume the length of the vehicle is 3.5 m and the size of pothole is about 0.5 m. If the average vehicle speed is 30 km/h, then in 0.5 s, the vehicle will cover approx. 4 m distance. For a total distance of 10 m and using a device sampling rate of 50 Hz, to collect 64 samples, it will take about 64 × 0.02 = 1.28 s. Therefore, we used a sliding window of 64 samples with overlap of 50% to divide the filtered continuous vibration sequence into labelled window segments that serve as the datasets required for the machine-learning model.

**Figure 10:**
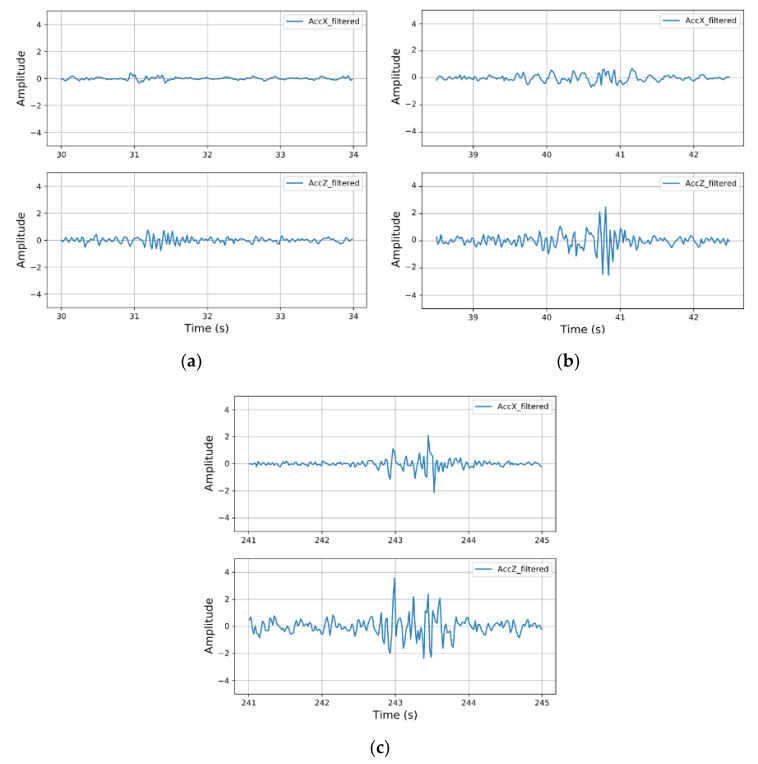
Acceleration signals along the *X*- (top row) and *Z*-axes (bottom row) of various types. (**a**) normal, (**b**) transverse and (**c**) pothole.

In this process, several threshold-based filters are also applied, to reject some nonpothole windows. First, we remove the windows with a speed lower than a specific threshold at which the pothole can hardly induce an abnormal vibration signal and be effectively detected by smartphones when the vehicle is running at a relatively low speed. Second, the RMS of the window is used for a preliminary judgement to determine whether the window is an anomaly, as the pothole causes more severe oscillations in the acceleration signal. Finally, some transverse windows are rejected using the *xz*-ratio filter [17], which is achieved by setting a proper threshold for $RMS_{x}/RMS_{z}$. In the experiment, there was strong vibration along the *X*-axis (in addition to the *Y*- and *Z*-axes) when the vehicle passed over a pothole with one side of its wheels. However, the vibration along the *X*-axis was significantly weaker when the wheels on both sides passed over the transverse road surface together. The smartphone accelerometer captured this difference, as shown in Figure 10b,c.

To locate the detected pothole, every potential pothole window that passes the preliminary screening is tagged with GPS coordinates. The ground-truth label is also tagged for subsequent model training and evaluation. Assuming an average vehicle speed of 30 km/h, then 0.5 s will cover approx. 4 m distance. Therefore, an anomaly (pothole or transverse) window is defined as a window that contains >25 anomaly samples (e.g., window c in Figure 9), whereas a window with no anomaly samples (e.g., window a in Figure 9) is labelled as ‘normal’. To avoid introducing uncertainty, windows containing fewer than 25 but more than 0 anomaly samples (window b in Figure 9) are discarded. Here, the anomaly sample means the sample belong to a tuple whose type is transverse or pothole.

Our practical implementation algorithm for selecting potential pothole windows is introduced in the Appendix A (Algorithm A3). According to the statistics for our experimental data, 75% of the normal windows can be removed using the speed and RMS threshold, while <1% of the pothole windows are lost. With the *xz*-ratio threshold, 50% of the transverse windows are removed, but 15% of the pothole windows are discarded. Therefore, the *xz*-ratio threshold is used in accordance with specific needs.

Using a sliding window and the three aforementioned thresholds, a total of 4088 potential windows were extracted from ‘poor’ and ‘bad’ road sections (as shown in Table 1). The extracted potential windows constituted the final datasets, as shown in Table 2. Each window contained acceleration data for three axes, and each axis had 64 samples.

### 3.3. Feature Extraction

Feature extraction generates valuable and significant features from raw data after transformation which has better discriminatory power between classes than raw data [30]. In traditional machine-learning, feature extraction is a challenging and critical step, as the user must thoroughly understand the application for training a model appropriately [31]. In many cases, sensor signals appear to be time sequences, for which digital signal processing has become a powerful feature-extraction method [32]. According to the literature [18,19,20,23], features are almost always extracted from the time domain, frequency domain, and wavelet domain in analyses of road vibration signals.

The performance of signals in the time domain, which intuitively reflects the vibration of the signals, is often investigated. Generally, researchers extract statistical variables such as the maximum value, minimum value, and variance as the features of the signal in the time domain [20,21,24]. In the present study, a statistical variable tuple (entropy, 5th-percentile value, 25th-percentile value, 75th-percentile value, 95th-percentile value, median, mean, standard deviation, variance, RMS, number of zero crossings, number of mean crossings) recommended by Ataspinar [33] was extracted from every window as time-domain features.

**Figure 11:**
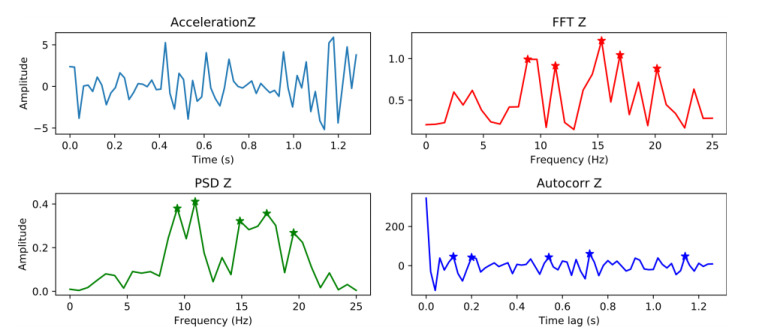
Acceleration signal along the *Z*-axis of a pothole, together with the results of applying the FFT, PSD, and autocorrelation function to it.

As mentioned by Ataspinar [34], the FFT, power spectral density (PSD), and autocorrelation functions provide useful information for vibration signal classification. We know that any complex random signal can be composed of a series of simple periodic signals. The FFT can decompose the signal and extract the periodic components by convoluting a series of sine waves having different frequencies with the signal [35]. The PSD function is useful for road condition analysis and was employed by Basavaraju et al. [24] and Sun [36]. The PSD function transforms a signal from the time domain to the frequency domain and provides its frequency spectrum, similar to the FFT. The difference is that the Power Density Spectrum focuses on describing the distribution of the signal spectrum energy; consequently, the height and width of the peak in the spectrogram are different. The autocorrelation function calculates the autocorrelation of the signal, that is, the degree of coincidence between the signal and the time-delayed version of itself. As Ataspinar [34] recommends, the abscissa and ordinate values of the peaks in the spectrum, which represent the frequency and magnitude of the components, respectively, can be used as features for the classifier. In this study, the five highest peaks resulting in 90 (3 transformations × 3 axes × 5 peaks × 2 values) features were selected for each window for analysis, as shown in Figure 11, which represented the main components of the signal.

**Figure 12:**
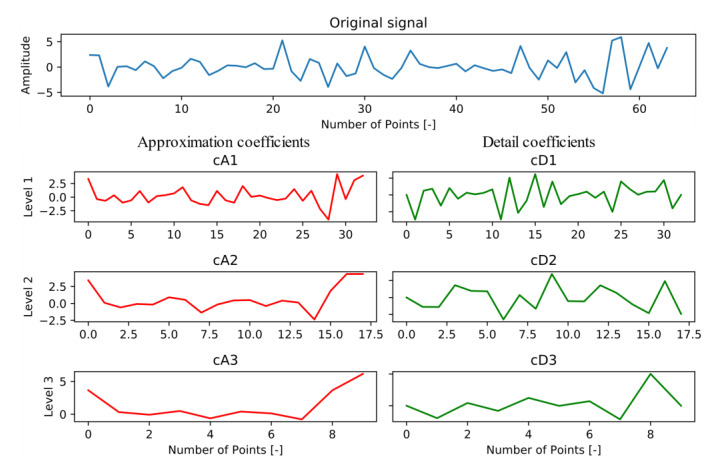
DWT of the rbio3.1 wavelet (levels 1 to 3) applied to an original acceleration signal.

The DWT convolutes the original signal with wavelets of different scales for decomposing the signal into several frequency subbands [37]. The advantage of wavelet transformation is that it has a certain resolution in both time and space, making it suitable for dynamic stochastic signal analysis. Another advantage is the large family of various mother wavelets to choose from; e.g., Mortlet, Haar, Symlets 5, Mexican Hat, Reverse Biorthogonal 3.1, and Daubechies 6 and 10 are used for road condition analysis [20,38,39,40,41]. This analysis method is based on the concept that different types of signals (normal, pothole, and transverse) have different frequency characteristics, which is confirmed by some frequency subbands generated from signal decomposition by DWT. Figure 12 shows a signal decomposed into the approximation and detail coefficients at three levels with the Reverse Biorthogonal 3.1 wavelet by pywt.dwt() in Python. The aforementioned statistical variable tuple was extracted from four scale subbands (cA3, cD3, cD2, and cD1), resulting in 144 (12 statistical variables × 4 subbands × 3 axes) features for every window.

### 3.4. Machine-Learning Classification

Machine-learning utilizes data or experience to automatically optimize the performance of computer programs [42,43]. Classification is one of the most important tasks in machine-learning. It involves using the model constructed with a training dataset to make predictions regarding the categories of items in the testing set. The object of this study was to use the vehicle vibration data collected by the smartphone to find potholes via machine-learning; a substantial classification task. Considering that traditional machine-learning algorithms already have satisfactory classification capabilities with relatively low requirements for datasets compared to Neural Networks, in this paper, traditional classifiers such as Logistic regression (LR), SVM and Random forest (RF) were applied to the features extracted from various domains of vibration signals, and their performance was evaluated.

The LR is a linear model for classification (rather than regression, despite its name) [44]. It uses a logistic function to map continuous input variables *X* to binary output values *Y*, in accordance with the hypothesis function:

(8) $$h_{\theta }\left(X\right)=\;\frac{1}{1+e^{-z}}\;=\;\frac{1}{1+e^{-\theta X}}$$

where *θ* represents the parameter matrix to be calculated through gradient descent for the cost function during the model training. LR is often used as a benchmark for comparison with other complex algorithms, because it is easily implemented, is efficiently trained, and yields good classification results. In this study, the LR function provided by Sklearn [45] was used with the default parameters.

The SVM is a supervised machine-learning algorithm that is widely used for solving classification and regression problems [46]. The idea of the SVM is to find a hyperplane that has the maximum margin in an *n*-dimensional space that distinctly divides the data points, as indicated by Equation (9). The kernel applied to the SVM can map data points to a higher-dimensional space, making nonlinear separation feasible. SVM is memory-efficient, effective in high-dimensional spaces, and versatile with various kernels that are effective for different scenarios [47]. Thus, it has been used by numerous researchers for road condition analysis [18,19,20,48,49,50,51,52,53,54]. In the present study, an SVM classifier with the radial basis function kernel was employed, with the help of the sklearn.svm.SVC function [55] provided by Sklearn for classification. All the other parameters were set to the default values.

(9) $$\underset{\omega ,b}{max}\frac{2}{\left\|\omega \right\|}\;s.t.y_{i}\left(\omega^{T}x_{i}+b\right)\geq 1,\;i=1,2,\ldots ,m.\;$$

The RF is an ensemble learning method for classification and regression that involves constructing multiple independently trained decision trees [56]. It is one of the most widely used machine-learning algorithms and exploits swarm intelligence by aggregating individual predictions based on various decision trees to decide the final classification. Generally, Bootstrap Aggregation (Bagging) is used in the ensemble procedure to reduce the variance of the decision trees, which enhances the stability of the prediction results [57]. In this study, we utilized the RF classifier [58] of the Sklearn library with n_estimators = 100 (100 decision trees in the forest).

### 3.5. Evaluation

To evaluate the performance of the aforementioned feature domains and algorithms, we adopted evaluation criteria from different aspects. Feature extraction is an essential step before classification—for both training and testing—and significantly affects the efficiency of the pothole detection system. Therefore, we compare the times required for feature extraction from different domains. However, the time required to train the model is less important, as the potholes are detected using the pretrained model deployed on the servers in real-world applications. To evaluate the performance of the features and classifiers, classification metrics were used in this study, including the precision, recall, and f1-score, which were derived from the confusion matrix based on the numbers of true positives (TP), true negatives (TN), false positives (FP), and false negatives (FN), as indicated by Equation (10). The precision corresponded to the correct rate of the detected potholes, the recall reflects the model’s ability to capture potholes from numerous road windows, and the f1-score identifies a balance between the precision and the recall. We also recorded the accuracy for the training set and testing set to evaluate the training effect of the model.

(10) $$\mathrm{precision}=\;\frac{\mathrm{TP}}{\mathrm{TP}+\mathrm{FP}};\mathrm{recall}=\;\frac{\mathrm{TP}}{\mathrm{TP}+\mathrm{FN}};f1-\mathrm{score}=\;\frac{2\times \mathrm{precision}\times \mathrm{recall}}{\mathrm{precision}+\mathrm{recall}};\;\mathrm{accuracy}=\;\frac{\mathrm{TP}+\mathrm{TN}}{\mathrm{TP}+\mathrm{FP}+\mathrm{TN}+\mathrm{FN}}$$

All operations were implemented on the Microsoft Windows 10 Professional OS, using an Honor notebook (Huawei, Shenzhen, China) with an Intel^®^ Core™ i5-8265U CPU @ 1.60 GHz and 16 GB RAM (Samsung, Seoul, Korea). All data processing was performed using Python 3.7.6, with machine-learning classifiers from Scikit-learn 0.22.1.

## 4. Results and Discussion

In this section, we discuss and evaluate the results with regard to the feature domain, classifier, and datasets. The details and parameters of the method were presented in the previous section. For each experiment, we randomly divided the dataset into training set and testing sets at a ratio of 70:30. All the experiments were performed 100× using the corresponding dataset, and the average value was taken as the final result. Since the goal of this paper is to detect potholes, most results shown in the paper are for potholes, ignoring the other two categories.

### 4.1. Feature Domain Evaluation

For feature extraction, the amount of time required and the pothole-detection accuracy must be considered. Therefore, we calculated the time taken to extract features from each window and the classification metrics for potholes. Owing to the stability and high accuracy of the RF algorithm, we utilized it to perform experiments on ‘All dataset’ (as mentioned in Table 2) with features extracted from different domains. The results are presented in Table 3.

According to the results in Table 3, the feature extraction from the frequency and time domains required significantly less time than that from the wavelet domain. Although the number of features extracted from the frequency domain was significantly larger than that for the time domain, the time required for the extraction was slightly shorter. This is because extracting the statistical variable tuple required significantly more time than detecting peaks. Extracting features from the wavelet domain required significantly more time than extracting features from the time and frequency domains. Additionally, even though the same statistical variable tuple was used, the amount of time required depended on the mother wavelet; db10 required the least time, and Haar required the most time (three times more than db10).

Regarding the pothole-detection accuracy, the features from the frequency and wavelet domains did not perform significantly better than those from the time domain in the experiments using our dataset; all the features achieved satisfactory results independently. By combining the features from the time and frequency domains, the strengths of each domain were exploited, i.e., the excellent precision of the frequency domain and high recall rate of the time domain.

### 4.2. Classifier Evaluation

We applied various classification algorithms, including LR, SVM, and RF, to ‘All dataset’, with features extracted from the time and frequency domains, as these were the most powerful features according to previous results. To evaluate the performance of these classifiers, the accuracy for the training and testing sets were considered, along with classification metrics for ‘normal’ and ‘pothole’. The results are presented in Table 4. The classification metrics for ‘transverse’ are not shown, because a few ‘transverse’ samples did not generate effective classification in our datasets.

As indicated by Table 4, all the algorithms exhibited good classification performance for ‘normal’, with both the precision and the recall rate exceeding 95%. As ‘normal’ samples accounted for most of the dataset, the three algorithms achieved comparable accuracy for the testing set. The RF achieved a relatively high accuracy for the training set because it had a more complex structure than the other two classifiers, particularly in the case of bagging with 100 decision trees. Among the classifiers that we investigated, the RF had the best comprehensive pothole-detection ability and exhibited the highest f1-score. The SVM had the highest classification precision for ‘pothole’, but its recall rate was slightly inferior to those of the other two classifiers; thus, it may miss moderate potholes. LR achieved a good precision and recall rate. Considering its low computing-power demand and good performance, it is suitable for cases with lower detection requirements and poor server performance.

### 4.3. Performance on Different Datasets

As mentioned in Section 3.2.5, we divided the data into two categories: the ‘Poor dataset’ and ‘Bad dataset’ (as shown in Table 2), which were extracted from urban and suburban roads, respectively. To verify the universality of the method, we applied RF with features extracted from the time and frequency domains to ‘All dataset’, ‘Bad dataset’, and ‘Poor dataset’. We also attempted to perform training on ‘All dataset’ and testing on ‘Bad dataset’ and ‘Poor dataset’ to evaluate the robustness of the approach. The results are presented in Table 5.

As shown in Table 5, the results of experiments 1 and 3 were excellent, with high accuracy in the testing set and high precision, recall and f1-score for pothole classification. The results of experiment 2 were satisfactory from the same perspective, considering that the dataset used was extremely small (it had only 69 potholes, as shown in Table 2). Thus, the proposed method is universal and does not rely on specific datasets. The results of experiments 3 and 5 were satisfactory, whereas those of experiment 1 were slightly inferior. Most of the positive samples (pothole) in ‘All dataset’ came from ‘Bad dataset’, which resulted in similar results among the three experiments. However, the testing set in experiment 1 included positive samples from the ‘Poor dataset’, leading to slightly inferior results.

Experiments 1, 4, and 5 involved training the model on the same dataset but testing it on different datasets. Experiment 4 obtained poor recall and f1-score for pothole classification, which was significantly worse than those of experiments 1 and 5, indicating that the robustness of the approach must be improved. To perform further analyses, both experiments 4 and 2 were tested on the ‘Poor dataset’; experiment 4 exhibited a slightly higher precision rate but a much lower recall rate than experiment 2. This was because in experiment 4, the model was trained on ‘All dataset’, which mostly contained samples from ‘Bad dataset’; thus, the trained model tended to detect severe potholes in suburban roads but missed moderate potholes in urban roads. By comparing the f1-score of the two experiments, we believe that the model trained on a mixed data set is not as good as training the model on a single data set. Thus, in real-life applications, training different models to adapt to different types of road segments can improve the pothole-detection ability of the system.

### 4.4. Discussion

**Figure 13:**
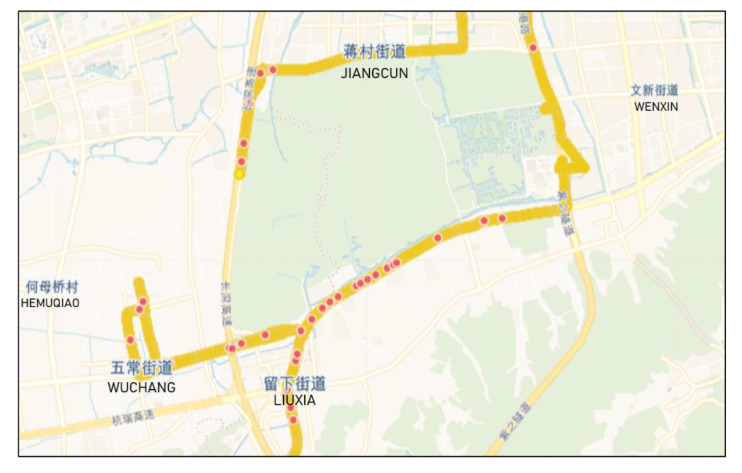
Partial map of Hangzhou. The yellow line indicates the trajectory of the vehicle and the red dots indicate the locations of the identified potholes.

The proposed method has been shown to detect potholes with good precision and recall. Figure 13 shows one of the driving trajectories of our experiments and the locations of potholes automatically detected using the proposed method. According to the results (Section 4.1, Section 4.2 and Section 4.3), feature extraction from the time and frequency domains is most effective, but extracting features merely from the time domain or frequency domain is suitable in cases where the computation must be limited. Generally, the RF algorithm has the best performance for pothole detection, but the SVM and LR are also appropriate for some special needs, for example when pursuing the precision of pothole detection or fewer calculations for classification. Experiments involving different datasets revealed that the proposed method is universal but not sufficiently robust for different types of roads. Therefore, broadly categorizing roads according to quality and training, the corresponding model is required for real-life applications. The robustness of this research, however, is not merely limited to road types; other factors, such as the smartphone and vehicle types, are also worthy of further study.

## 5. Conclusions

The objective of this study was to develop a method for using smartphones with embedded accelerometers to identify potholes in road surfaces, which would significantly reduce the time and resources required for pothole detection. The feasibility of this idea was confirmed, as machine-learning models with features extracted from acceleration signals along three axes achieved significant precision and recall for pothole classification. Although the research was conducted offline, the designed pipeline can be migrated online and applied in practice. It is predicted that the pothole-detection accuracy will be further improved with the increase in data after migration online.

In this study, a complete data-processing workflow for road pothole detection using smartphones was designed, and novel concepts were introduced. Segmenting a continuous signal sequence with a sliding window utilizing appropriate thresholds can remove a large amount of irrelevant data. Additionally, <1% of the important data related to potholes are missed with the speed and RMS thresholds. Evaluating the results with regard to various aspects revealed that the RF classifier with features extracted from the time and frequency domains had the best comprehensive performance among the methods tested. The universality of the proposed method was confirmed by applying it to datasets generated from different types of roads. Unfortunately, the robustness was revealed to be insufficient when the trained model was tested on another dataset. This indicates that different models must be trained for different road types.

For real-life applications, data processing (except for labelling) can be done on the client side (i.e., the smartphone), with potential potholes being selected using the threshold method during the segmentation procedure, and real potholes being distinguished from false potholes on the server side using the pretrained machine-learning classifiers. This approach conserves the phone battery power and limits the extent of mobile communication data, as only potential windows will be uploaded. Additionally, it avoids continuous reliance on a mobile network connection; hence, the application continues to function even when some parts of the road have poor cellular service. Another difference is that labelling would not be completed, for two reasons: (1) app users find irritating and feel that it is dangerous and impractical to perform labelling while driving and (2) labelling is not needed, because potholes are identified automatically using the pretrained models on the server side.

This study had limitations that must be addressed in future research. The dataset that we used for training was relatively small, which led to two problems. First, it limited the selection of models; for example, the neural network was rejected, as it required a large dataset to generate features by itself. Second, it limited the improvement of model accuracy. Crowdsourced data would enable a significant improvement to the system through the use of swarm intelligence to cluster the data. Another problem of the dataset is that the disproportional distribution of samples among the three categories introduced a bias that may have affected the individual precision and recall rate of classification. Specifically, ‘transverse’ road surfaces were not correctly identified, owing to the small number of samples. Various data-acquisition conditions, e.g., the type of vehicle, payload, running speed, placement of the smartphone, and position in the vehicle where the smartphone is placed, affect the performance of the system. One promising area that could be pursued to improve the vibration-based pothole detection method is to incorporate a vision-based component to improve the vibration-detection method. Since the collected data is associated with users’ privacy information (e.g., driving trajectories, video footage), certain anonymization and encryption technologies would be needed, which is worthy of further study. In summary, all the aforementioned limitations are potential research topics that need to be addressed in future studies.

## Figures and Tables

**Table 1:** Road type, quality and distance of the experimental road sections.

Road Type	Road Quality	Distance (km)
National motorway roads	Good	51.8
Urban roads	Poor	29.4
Suburban roads	Extremely bad	25.7

Note: The road quality here is not strictly quantified, but a relative concept felt by the observer.

**Table 2:** Three final datasets of potential windows.

Datasets	Number of Normal Windows	Number of Pothole Windows	Number of Transverse Windows	Total Number of Windows
Poor	817	69	13	899
Bad	2784	405	0	3189
All (poor and bad)	3061	474	13	4088

**Table 3:** Performance of different feature domains with the RF classifier applied to ‘All dataset’. The precision, recall, and f1-score correspond to ‘pothole’.

Features		Number of Features for Each Window	Time Required (ms)	Precision	Recall	F1-Score
Time domain		36	2.50	0.878	0.741	0.804
Frequency domain		90	2.27	0.884	0.724	0.796
Time and frequency domain		126	4.77	0.885	0.750	0.812
Wavelet domain	Reverse biorthogonal 3.1	144	12.06	0.881	0.739	0.804
	Haar		17.05	0.883	0.736	0.803
	Symlets 5		7.49	0.871	0.727	0.792
	Daubechies 6		7.51	0.876	0.713	0.786
	Daubechies 10 (db10)		5.09	0.879	0.735	0.801

**Table 4:** Performance of different classifiers with features extracted from the time and frequency domains for ‘All dataset’.

Classifiers	Accuracy for Training Set	Accuracy for Testing Set	Window Type	Precision	Recall	F1-Score
LR	0.961	0.952	normal	0.965	0.984	0.974
			pothole	0.851	0.734	0.788
SVM	0.951	0.948	normal	0.952	0.992	0.971
			pothole	0.908	0.642	0.752
RF	0.999	0.957	normal	0.965	0.988	0.976
			pothole	0.885	0.750	0.812

**Table 5:** Performance of the RF classifier with features extracted from the time and feature domains applied to different datasets. The precision, recall, and f1-score correspond to ‘pothole’.

Experiment ID	Training Set	Testing Set	Accuracy on Training Set	Accuracy on Testing Set	Precision	Recall	F1-Score
1	All	All	0.999	0.957	0.885	0.750	0.812
2	Poor	Poor	0.999	0.935	0.686	0.460	0.551
3	Bad	Bad	0.999	0.965	0.897	0.813	0.853
4	All	Poor	0.999	0.928	0.826	0.260	0.396
5	All	Bad	0.999	0.965	0.893	0.821	0.856

## Appendix A

**Algorithm A1.** Using the gravity to align the Z-axis of the acceleration data with that of the vehicle.
Update *α* and *β* every 2 min using the steadiest window (if not found, choose the latest *α* and *β*): Extract windows of 6 s using sliding windows with a step of 2 s; Find the steadiest window by choosing the window with the smallest standard deviation; Utilize the mean value of the 3 axes of the selected window to calculate Euler angles *α* and *β* via Equations (3) and (4); Complete the transformation for the entire 2-min window using Equation (5);

**Algorithm A2.** Utilizing braking or acceleration events to align the X- and Y-axes of the acceleration data with the vehicle.
Update *γ* every 2 min using a breaking or acceleration event (if not found, choose the latest *γ*): Extract windows of 6 s using sliding windows with a step of 2 s; Find the most representative window as a breaking or acceleration event: Calculate the Δ*V* and Δ*Z* ($\Delta V=\;V_{max}-V_{min}$, $\Delta Z=\;Z_{max}-Z_{min}$) of each window; A window that satisfies Δ*V >* 5, Δ*Z <* 1.5, and Δ*V* − Δ*Z >* 4 is treated as an event; **If** more than one event is found, choose the one with the largest Δ*V* – Δ*Z* as the most representative one;
**If** a representative window does not exist, **return** to the first step; else, find the 5 rows of sample data with the largest horizontal acceleration component $\sqrt{{a^{\prime }}_{x}^{2}+{a^{\prime }}_{y}^{2}}$ and calculate their mean value $\bar{a_{x}^{\prime }}$*,* $\bar{a_{y}^{\prime }}$; Calculate γ using Equation (6); Complete the transformation for the entire 2-min window using Equation (7);

Note: *V* represents the velocity of the vehicle, and *Z* represents the acceleration of the *Z*-axis. The specific thresholds in the algorithm are summarized from many attempts depending on our datasets.

**Algorithm A3.** Selecting potential windows.
Segment continuous signal into labelled windows of 64 samples using sliding windows with 50% overlap: **If** the mean *speed* of a window is <3 m/s, **return** the first step; else, proceed to the next step; **If** the sum of the *RMS* values along the 3 axes of a window is <1.2 m/$s^{2}$, **return** to the first step; else, proceed to the next step; **If** $RMS_{x}/RMS_{z}\;$< 0.25, return to the first step; else, proceed to the next step; Tag the window with GPS coordinates; Tag the window with the ground-truth label: **If** a window contains no anomaly samples, tag it with the ‘normal’ label; **Else**, **if** the window contains >25 anomaly samples, tag it with the corresponding anomaly label (‘pothole’ or ‘transverse’); **Else**, **return** to the first step;

Note: The specific thresholds in the algorithm are summarized from many attempts depending on our datasets.
